# A Case for Dynamic Percolation Underlying Mechanistic Crossovers in the Relaxation of Liquids

**DOI:** 10.1021/acs.jpcb.5c01033

**Published:** 2025-06-17

**Authors:** Marcus T. Cicerone, Jessica Z. Dixon, John P. Stoppelman, Kelly Badilla-Nunez, Jesse G. McDaniel

**Affiliations:** † Department of Chemistry and Biochemistry, 1372 Georgia Institute of Technology 950 Atlantic Drive, Atlanta, Georgia 30332, United States; ‡ 12381 Institute of Chemistry, Academia Sinica, Taipei 115, Taiwan; § School of Chemical and Biochemical Engineering, Georgia Institute of Technology 900 Atlantic Drive, Atlanta, Georgia 30332, United States

## Abstract

One of the outstanding questions regarding liquid dynamics is the cause of the apparent mechanistic changes in relaxation at the characteristic temperatures *T*_A_ and *T*_B_, where, as the temperature is lowered, α relaxation times become super-Arrhenius, and the β_JG_ relaxation bifurcates from α, respectively. Based on system-averaged picosecond-time scale dynamic signatures in five molecular liquids and a Kob-Andersen (KA) model system, we propose that these mechanistic changes arise from the percolation of distinct dynamic environments in the liquid, where the dynamic environment of a particle is defined by the number of structural excitations in its first solvation shell. Analysis of the KA system trajectories supports this idea and suggests that the most prominent effects can be understood in terms of environments that are mobile or immobile on a picosecond time scale. Further, the existence and percolation of these dynamic environments can account for many of the characteristic dynamic signatures of glass-forming liquids.

## Graphical abstract

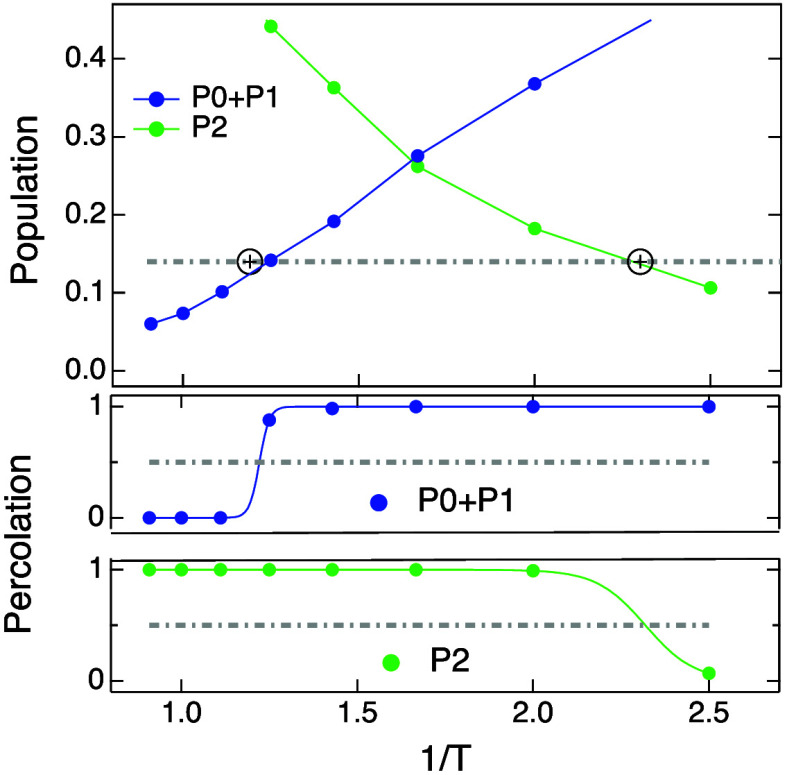

## Introduction

Relaxation phenomena in liquids reveal the presence of dynamic heterogeneity, temperature-dependent mechanistic crossovers, and, ultimately, ergodicity breaking at the glass transition. Perhaps the most striking of these is the strongly super-Arrhenius behavior of primary (α) relaxation or viscosity. Goldstein^1^ proposed that neighbor-induced dynamic constraints in liquids could produce this behavior below some onset temperature. Johari and Goldstein later identified a secondary (β_JG_) relaxation process^2^ that distinctly separates from α relaxation at a second, lower temperature and appears to signal yet another mechanistic onset.^3^

Figure 1 exemplifies dynamic behavior for propylene carbonate (PC) at these onset temperatures. The higher onset temperature is often associated with the point (*T*_A_) where the primary relaxation time (τ_α_) crosses over from Arrhenius behavior, τ_α_ ∝ Exp­[*E*_A_/*k*_B_ *T*], *E*_A_ = constant for *T* > *T*_A_, to super-Arrhenius behavior. Below *T*_A_, liquids evince nonergodicity on the time scale of τ_α_, in other words, dynamic heterogeneity,^4^ which can be seen through the time dependence of the rotational relaxation function *c*_R_(*t*) ∝ Exp­[(−*t*/τ)^β_KWW_^]. As shown in the inset to Figure 1, β_KWW_ generally begins to decrease from its high-temperature asymptotic value near *T*_A_, signifying a broadening in the distribution of dynamic environments that persist on the time scale of τ_α_. At the same time, *E*_A_ takes on a distinct temperature dependence—quadratic for PC and other liquids,^3,5^ evinced by the linear behavior of *E*_A_^1/2^ vs 1/*T* in Figure 1.

**Figure 1:**
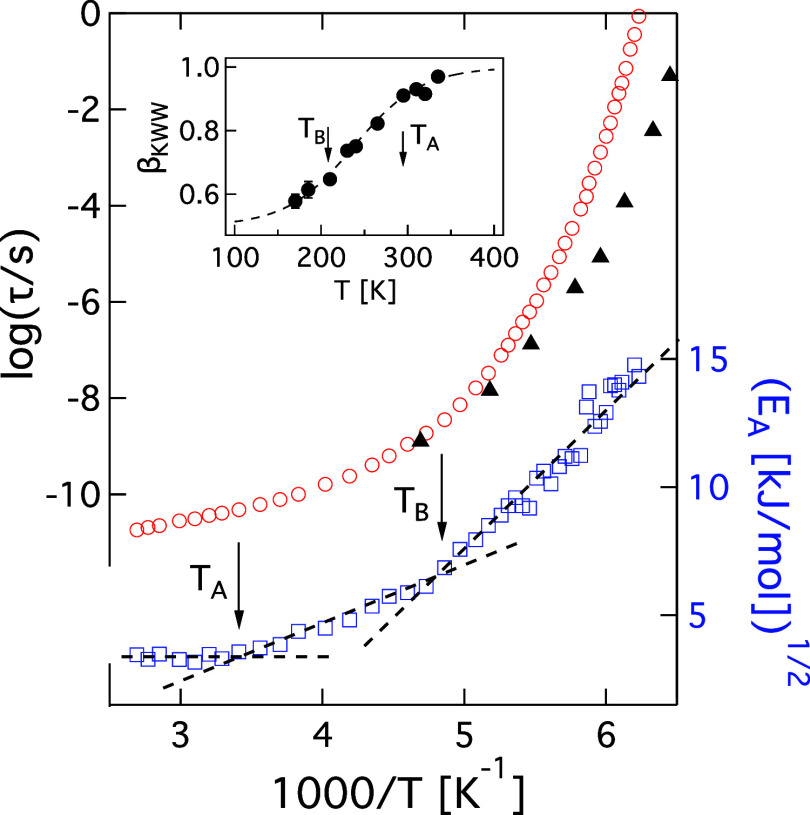
Relaxation and transport data for propylene carbonate. (Left Axis) Red circles—α and black triangles—β_JG_ relaxation times from dielectric spectroscopy.^10^ (Inset) The stretching exponent β_KWW_ as a function of Temperature.^11^ (Right Axis) *E*_A_ values obtained from relaxation data as [*d* log­(τ_α_)/*d*(1/*T*)]^1/2^. Dashed lines are guides to the eye. The inset is reproduced from ref 11 with permission from the Royal Society of Chemistry.

Evidence for another mechanistic onset is found at a lower temperature,^3,6^ often denoted as *T*_B_. As shown in Figure 1, *E*_A_ in PC takes on a stronger quadratic temperature dependence^3^ at *T*_B_, and relationships between transport coefficients such as τ_α_ and the diffusion coefficient (*D*_T_) change abruptly.^7,8^ Additionally, β_KWW_ values approach their asymptotic minimum, and there is evidence for the emergence of a dynamically inactive, solid-like phase.^5,6,9^ Finally, at *T*_B_, the β_JG_ relaxation^2^ appears to bifurcate from the α process.

Stillinger^12^ and others elaborated on Goldstein’s dynamic constraints proposal,^1^ creating a potential energy landscape (PEL) formalism that has been used to frame considerations of liquids and glass. In this framework, dense, amorphous packing leads to locally favored particle configurations referred to as inherent states (IS). These lie in energy basins with distributions of width, depth, and connectivity that encode the thermodynamic properties of the liquid. Dynamic properties of the liquid arise through interbasin (IB) transitions. Chandler proposed that, below *T*_A_, IB transitions must be associated with structural excitations,^5^ localized, activated particle configurations that facilitate particle rearrangements and correspond to population at the top of interbasin barriers on a PEL. In simulations,^13,14^ these excitations are identified by ∼1 ps time scale interbasin barrier crossing events (hops) that occur as excited particle configurations pass over a dividing surface between two basins and rapidly rearrange into a new inherent state. It is important to note that, while excitations are identified by dynamic events, they are themselves not dynamic but thermodynamic, structural entities.

If all dynamic and thermodynamic information is encoded in the PEL, then the underpinnings of the mechanistic crossovers at *T*_A_ and *T*_B_ must have their origin in structures and dynamics that can be identified on a picosecond time scale since that is the shortest time on which basin occupation can be determined^15^ and the natural time scale for interbasin barrier crossing events.^13,14,16^ In support of this notion, it has long been recognized that picosecond motions encode slower dynamics.^17^ Below, we find evidence from picosecond-time scale excitations (populations at the top of interbasin barriers) at *T*_A_ and *T*_B_ leading us to postulate that the changes in relaxation mechanism at those temperatures are due to the percolation of distinct dynamic environments that are defined by the number of excitations in a particle’s first solvation shell. We then test this hypothesis by analyzing trajectories of the Kob-Andersen (KA) model system, finding support for and refining this idea.

## Methods

### QENS Data Acquisition and Reduction

The acquisition of QENS data analyzed here has been reported previously.^16,18^ In summary, the QENS data was acquired with the Disk Chopper Spectrometer (DCS) installed at the NG4 guide at the NIST Center for Neutron Research.^19^ The neutron wavelength was set at 4 Å, corresponding to a momentum transfer (*q*) range of (0.2 to 2.8) Å^–1^, and data were binned at steps of 0.1 Å^–1^. The instrumental resolution was 0.19 meV, and the maximum energy transfer was 4.5 meV. Data at each temperature was obtained within (3 to 6) h.

Experiments were performed on cooling from the high temperature to avoid potential artifacts from crystal nucleation. We ensured no sign of crystallization even on reheating when crystal nuclei would have grown if they had been seeded at low temperatures. The absence of crystallization was confirmed by noting an absence of Bragg peaks in plots of scattered intensity vs *q*.

The data were corrected for (i) background from an empty can, (ii) dark count background with no neutron flux, and (iii) detector efficiencies by measuring a vanadium standard. All the data files were reduced using the DAVE software available at www.ncnr.nist.gov/DAVE. Instrument resolution was estimated as a Gaussian from sample scattering at 30 K.

QENS data were analyzed in the frequency domain and in the time domain after transformation. *S*(*q*,ω) was directly transformed to the time domain *F*(*q*,*t*) using DAVE software. Noise is propagated in the Fourier transform operation, and additional errors can arise through truncation and course sampling intervals. These factors are considered and standard uncertainties are calculated by the DAVE software. Errors arising were less than 0.1% of *F*(*q*,*t*) values. The real part of the Fourier transformed data was used to calculate *F*(*q*,*t*), and no filtering options were used as they were not found necessary.

### Simulations of the Kob-Anderson Model

Kob-Andersen (KA) binary LJ simulations were performed with 10,000 particles at the standard choice of 80%/20% A/B particles and reduced density ρ = 1.2. In the KA mixture, the A/B particles interact with LJ pair potentials given by σ_BB_/σ_AA_ = 0.88, σ_AB_/σ_AA_ = 0.8, ϵ_BB_/ϵ_AA_ = 0.5, and ϵ_AB_/ϵ_AA_ = 1.5. KA simulations were run with the OpenMM software, utilizing a Langevin thermostat and pairwise interactions cutoff at *r*_cutoff_ ≈ 3σ_AA_. The integration time step was set as Δ*t* = 0.0025τ, with τ being the reduced time unit of the KA model.^13^ Simulations were performed at eight different temperatures spanning the range *T* = 0.4–1.1 in reduced temperature units; this range spans temperatures just below *T*_B_ to temperatures above *T*_A_ (Table 1). Each system was equilibrated for ∼ 10^3^τ, which is sufficient given they were started from the target system density ρ = 1.2. Because the primary analysis of each simulation is the computed self-intermediate scattering function (*vide infra*), simulation trajectory lengths and output frequency were chosen to provide sufficient resolution/statistics at both short/long time scales. Trajectory snapshots were saved at every 0.05τ time point. The specific trajectory length for each temperature simulation is given in the Supporting Information; trajectories spanned in length from 10^3^–10^4^τ, with longer trajectories generated for the lower temperature simulations.

**Table 1:** Characteristic Temperatures

molecule	*T*_A_ [K]	*T*_B_ [K]	*T*_A_?^e^ [K]	*T*_B_?^e^ [K]
propylene	291^a^	209^a^		
carbonate	294 ^3^	200 ^3^		
		187 ^20–22^		
		182 ^23^		
		196 ^24^		
		210^b^		
average	292 ± 2	195 ± 10		
propylene glycol	321 ^25^			280 ^26^
	292 ^21^			239 ^27^
				251 ^21^
				248^b^
average	305 ± 20			255 ± 18
glycerol		310 ^28^	338 ^25^	
		288 ^29^	330 ^30^	
		285 ^26^	288 ^21^ ^d^	
		295 ^30^		
		283 ^31^		
average		294 ± 13	334 ± 6	
ortho terphenyl	455 ^32^	290 ^26,28,32,33^	341 ^25^	
		285 ^34^	357 ^25^	
		293 ^35^	340 ^25^	
		291 ^36^		
		279^b^		
average		288 ± 5	346 ± 10	
sorbitol		340 ^30^		
		351^b^		
average		345 ± 8		
Kob-Andersen	0.84 ± 0.09^c^	0.435 ^37^		
average	0.84 ± 0.09	0.435		

a *T*_A_ and *T*_B_ obtained from Stickel^3^ analysis of data of Leukenheimer et al.^27^

b *T*_B_ values estimated as the temperature where α and β relaxation processes merge.

c From this work. Temperatures for KA are given in reduced units.

d This temperature was designated at *T*_A_ in ref 21 but corresponds to *T*_B_ as determined by other authors, so it is not included in the average for *T*_A_.

e These values do not fall at *T*_A_ or *T*_B_ by our analysis, but at a solidification temperature, as explained in Results.

The self-intermediate scattering function *F*_s_(*q*,*t*) was computed from the KA simulations at the different temperatures, using the formula

1 $$F_{s}\left(q,t\right)=\frac{1}{N}\left\langle \sum_{i=1}^{N}\exp\left(iq\cdot \left(r_{i}\left(t\right)-r_{i}\left(0\right)\right)\right)\right\rangle$$

The self-intermediate scattering function computed from the KA simulations, in tandem with the corresponding experimental scattering functions obtained from QENS experiments on the molecular liquids, are the primary dynamical data upon which the subsequent analysis of excitations is based.

### Determining Characteristic Temperatures

Table 1 displays characteristic temperatures *T*_A_ and *T*_B_ obtained from literature sources and calculations herein. The *T*_A_ values from literature are all determined through a change from Arrhenius to super-Arrhenius temperature dependence of τ_α_. Some literature values listed under *T*_B_ were obtained from a change in the temperature dependence of τ_α_, usually by plotting its derivative as in Figure 1.^3,26,30–32,36^ The remaining *T*_B_ entries from published literature^20–24,27,28,33–35^ were determined as the mode-coupling critical temperature (*T*_c_) by fitting material relaxation to a form: τ_α_(*T*) ∝ (*T* – *T*_c_)^−γ^, or finding the temperature at which nonzero solutions for the mode-coupling nonergodicity factor *f*(*k*) first emerge.^37^ Values found as *T*_B_ or *T*_c_ are statistically indistinguishable.

Entries marked with letter superscripts (a–c) were determined by us. In cases marked with a superscript *a*, characteristic temperatures were determined using the method of Stickel et al.^3^ and published relaxation data^10^ as shown in Figure 1. For entries with a *b* superscript, we estimated *T*_B_ as the temperature where τ_α_ and τ_β,JG_ bifurcate. For sorbitol, this was done using τ_β,JG_ from Fujima et al.^38^ to generate a second *T*_B_ data point. As a check on this approach, we performed a similar analysis for PC and OTP, where *T*_B_ values are well-established. *T*_B_ determined in this way agreed reasonably well with *T*_B_ and *T*_c_ from literature. The single entry marked with a *c* superscript is *T*_A_ for the KA model, which we determined from the onset of super-Arrhenius temperature dependence of α relaxation times. We found τ_α_ as the time of the 1/e point of *F*_*s*_(*q*,*t*) at the peak of the structure factor, *q*_*max*_, for A particles. The τ_α_ vs 1/*T* data from which *T*_A_ is derived is shown in the Supporting Information.

We now call attention to the last two columns in Table 1, labeled *T*_A_? and *T*_B_?. Based on an analysis we will present in Figure 3 below, these values for *T*_A_ and *T*_B_, taken from literature, fall at a what we suspect is a temperature intermediate between actual *T*_A_ and *T*_B_ temperatures where we see evidence for percolation of microscopic regions in the liquid that are solid-like on a picosecond time scale.

### Coordination Numbers

Coordination numbers (*z*) used to construct Figures 3 and 4 are given in Table 2 and were calculated from simulated center-of-mass radial distribution functions (*g*(*r*)) according to

2 $$z\left(r_{\min}\right)=4\pi \rho \int_{0}^{r_{\min}}g\left(r\right)r^{2}dr$$

where ρ is the average density, and *r*_min_ is the first minimum in *g*(*r*). The KA simulations utilized to compute *g*(*r*) have been described in the Methods Section, whereas simulations utilized to compute *g*(*r*) for glycerol, OTP, sorbitol, and propylene carbonate are described in the Supporting Information. We note that all other data/analyses of these molecular liquids are based on the experimental QENS data, and the simulations are used for no purpose other than computing coordination numbers.

**Table 2:** Coordination Numbers

molecule	*T* [K]	*z*
propylene carbonate^a^	200	12.5
	220	12.4
	240	12.4
	260	12.4
	280	12.5
	300	12.4
	**Value Used**	12.4
propylene glycol^39^	298	13
	**Value Used**	13
glycerol^40^	300	13.1
	350	13.6
	400	13.0
	450	13.2
	**Value Used**	13.3
ortho terphenyl (LW)^a^	275	10.9
	300	10.8
	400	10.5
	500	10.2
ortho terphenyl (atomistic)^41^	267	13.3
	**Value Used**	11.5
sorbitol^a^	373	12.9
	**Value Used**	12.9
Kob-Andersen^a^	1.0	11.3
	**Value Used**	11.3

a This work.

### Identifying Excitations

We are interested in quantifying the population of particles involved in structural excitations at the tops of the interbasin barriers. Excitations are identified in simulations^13,14^ by picosecond-time scale particle rearrangements over length scales on the order of 20% of a particle radius.

Signatures of inherent state and interbasin motion are also found in experimental quasielastic neutron scattering (QENS) data. A decade ago, we showed^18^ that, at 1 ps, when diffusion is not important, the self-intermediate scattering function (*F*_s_(*q*,*t*)), the Fourier transform of the scattering function *S*(*q*,ω) from QENS data of five molecular liquids could be fit without significant residuals with a two mode function (See Figure 2A)

3 $$F_{s}\left(q,t\right)=\left[1-\Phi \left(t\right)\right]e^{-{\left(q\pi \sigma_{IS}\right)}^{2}}+\Phi \left(t\right)e^{-{\left(q\pi \sigma_{IB}\right)}^{2}}$$

where σ_IS_ and σ_IB_ are distinct length scales of localized motion and Φ­(*t*) is the time and temperature-dependent fraction of particles executing the larger length scale motion. The strong temperature dependence of σ_IS_ and weak temperature dependence of σ_IB_ found in that work are respectively consistent with increased length scale intrabasin exploration at elevated temperatures and a constant length scale for particle rearrangements associated with interbasin barrier crossing. At all temperatures, σ_IB_ ≥ 3σ_IS_ for each material. The well-separated length scales make it trivial to separate these variables in the fitting process. The σ_IB_ value dominates the slope in Figure 2A at *q* ^2^ < 1, below the structure factor peak at *q* ≈ 1.4 Å^–1^, whereas σ_IS_ dominates the slope at *q* ^2^ > 2.

**Figure 2:**
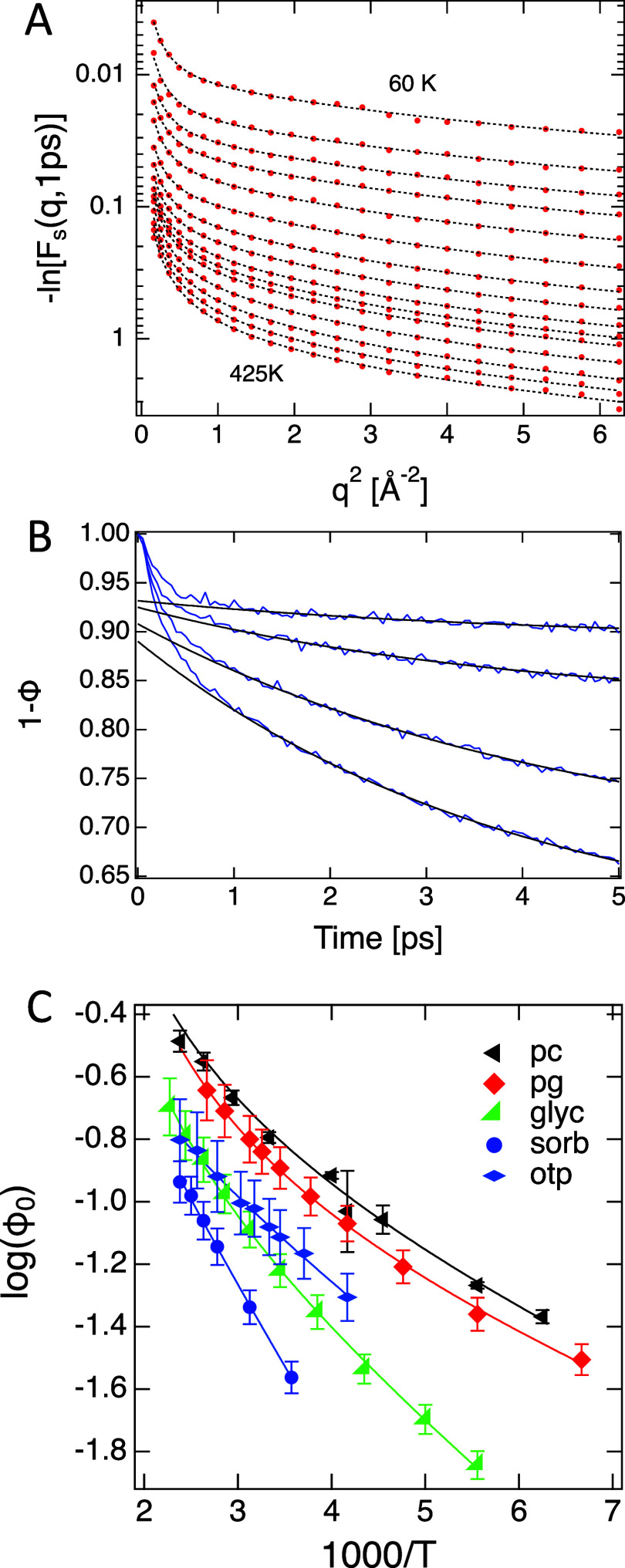
(A) Fits of eq 3 to *F*_s_(*q*,*t* = 1 ps) of propylene glycol at temperatures in the range (60 to 425) K. (B) (1 – Φ_0_) from the KA system at *T* = [0.4, 0.5, 0.6, 0.7]. (C) Instantaneous excitation populations, Φ_0_ as a function of inverse temperature for propylene carbonate (pc), propylene glycol (pg), glycerol (glyc), sorbitol (sorb), and ortho terphenyl (otp). Solid lines are guides to the eye.

Vispa et al.^42^ subsequently used Bayesian inference to evaluate models of *S*(*q*,*E*) from QENS data of glycerol to establish the unambiguous presence of three distinct dynamical processes with Lorentzian form; translational diffusion and two additional localized modes. The fast and slow localized modes had time scales of 100 fs and 1 ps and distinct length scales. In their model, they proposed that particles could participate in either fast or slow motion, but not both.

We subsequently performed a Bayesian model analysis for *S*(*q*,*E*) and *F*_s_(*q*,*t*) of propylene carbonate, also finding a three-Lorentzian model to provide the best fit. In a slight departure from the approach of Vispa et al., we proposed a heterogeneous model where all particles undergo fast localized motion, overdamped vibrations around the inherent state configurations, but only some particles participate in slightly slower and larger-scale interbasin transits, consistent with a PEL picture, giving the following postulated form for *S*(*q*,*E*).

4 $$S\left(q,E\right)=\left(1-\mathrm{\Phi ̃}\left(E\right)\right)L_{D}⊗\left[\left(1-a_{v}\right)\delta \left(E\right)+a_{v}L_{v}\right]+\mathrm{\Phi ̃}\left(E\right)L_{D}⊗\left[\left(1-a_{v}\right)\delta \left(E\right)+a_{v}L_{v}\right]⊗\left[\left(1-a_{h}\right)\delta \left(E\right)+a_{h}L_{h}\right]$$

where *L*_*i*_ = Γ_*i*_π^–1^(*E* ^2^ + Γ_*i*_^2^)^−1^, ⊗ is the convolution operator, the convolutions are over frequency (energy). Γ_D_ = *D*_T_ *q* ^2^, where *D*_T_ is the diffusion coefficient, and *a*_*IS*_ and *a*_IB_ are respectively *q*-dependent scattering amplitudes from vibration of inherent states and transits (hopping) over interbasin barriers. Both terms in eq 4 include diffusive (*D*) and inherent state (IS) vibrational motion. In agreement with Vispa et al., we found that the two localized modes had Gaussian distributions of displacements following the functional form proposed by Rahman:^43^

5 $$a_{i}\left(q\right)=c_{i}\left\{1-\exp\left[-{\left(\pi \sigma_{i}q\right)}^{2}\right]\right\}$$

where the σ_*i*_ represent the characteristic length scales of motion for mode *i*.

The Fourier transform of eq 4 gives the time-domain representation as

6 $$F_{s}\left(q,t\right)=e^{-t/\tau_{D}}\left[1+a_{IS}\left(e^{-t/\tau_{IS}}-1\right)\right]\times \left[\left(1-\Phi \left(t\right)\right)+\Phi \left(t\right)\left(1+a_{IB}\left(e^{-t/\tau_{IB}}-1\right)\right)\right]$$

where τ_*i*_ = ℏ/Γ_*i*_. On a picosecond time scale when *t*Γ_D_ < ℏ < *t*Γ_IB_ ≤ *t*Γ_IS_, eq 6 reduces essentially to eq 3.

In this later work, we noted that the time-dependence of Φ­(*t*), σ_IS_, and σ_IB_ were also consistent with motion on a PEL lansdscape.^16^ σ_IS_(*t*) increased monotonically, then plateaued, consistent with bounded exploration of a basin. σ_IB_(*t*) first increased, then decreased in time, consistent with hopping over a barrier, and subsequently some particles hopping back to the initial basin. We confirmed this origin for the nonmonotonic behavior of σ_IB_ through atomistic MD simulation.^40^ Additional fits of eq 3 to experimental *F*_s_(*q*, *t*) data, as well as time-dependent σ_IS_ and σ_IB_ from fits to eq 6 are shown in Supporting Information.

The signature of the excitation population is found in Φ­(*t*). This parameter gives the fraction of particles that have been involved in an excitation-mediated IB barrier crossing up to time *t*. As shown in Figure 2B, (1 – Φ­(*t*)) drops quickly at first but then decays more slowly. This rapid drop is a result^40^ of the same rapid hopping motion that is used in simulation to identify excitations, and the amplitude of the drop gives us the fraction of particles involved in excitations. The 1 ps time scale at which this rapid drop occurs is the time that σ_IB_ reaches a local maximum approximately equal to 20% of the hydrodynamic radius (see Supporting Information). This time and length scale are consistent with criteria applied in particle tracking for identifying excitations through individual particle displacements.^13,14,44^

Given the above, we take Φ­(1 ps) = Φ_0_ at the population of particles in excitations, at the top of interbasin barriers. Figure 2C shows Φ_0_ derived from QENS data for the five liquids. The solid lines are guides to the eye, but we note a positive deviation from Boltzmann behavior at low temperatures for most of the liquids. Assuming Boltzmann statistics apply, Φ_0_ = Exp­[−*E*_Φ_/*k*_B_ *T*]/*Z*, and the deviation from behavior at *T* > *T*_B_ could be due to temperature-dependent changes in the partition function, *Z*. Middleton et al.^45^ found distinct groups of interbasin energy barriers for several model systems. It is possible that one such grouping begins to become unavailable below *T*_B_, reducing *Z* and leading to the non-Boltzmann behavior.

In the analysis of simulation data we follow the approach of others,^13,14^ assigning particles to excitations when they exhibited a sufficiently large displacement *δr* ^2^ over a time window *δt*:

7 $$\delta r^{2}\left(t\right)={\left|\mathrm{r⃗}\left(t\right)-{\mathrm{r⃗}}_{\mathrm{avg}}\left(t-\delta t\right)\right|}^{2}$$

where *r⃗* represents the particle’s position and *δt* = 1.3 ps. We use Φ_0_ determined from eq 3 constrain the number of particles assigned to excitations through a displacement criterion, *δr⃗*_min_ so that the fraction of particles with *δr⃗* ≥ *δr⃗*_min_ is equal to Φ_0_.

## Results

### Evidence for Distinct Dynamic Environments

All the systems we have analyzed have Φ_0_ ≈ 0.05 at *T*_B_, leading us to search for a potential mechanistic connection. Initially, we explore the hypothesis that the number of nearby excitations presents distinctly different dynamic environments. Retaining the assumption of facilitated dynamics, we argue that only three distinct environments are relevant: those having zero, one, or at least two excitations in their immediate vicinity. We indicate populations in these environments as *P*_0_, *P*_1_, and *P*_2_.

**Why three environments?** To keep the argumentation simple, we assume the following: (i) significant interbasin barriers exist and excitations mark population at a saddle point of the barrier, (ii) each saddle point between IS basins has only one primary reaction coordinate (a nonbifurcating minimum surface) and that the curvature at the top of the barrier is symmetric about the maximum. In this case, each excitation presents a binary choice between two inherent states for the system, each with the same probability. For convenience, we will define *IS*_*A*,*j*_ and *IS*_*B*,*j*_ respectively as the initial inherent state occupied by and the new inherent state available to the particles involved in excitation *j*.

In our idealized scenario, particles in *P*_0_ environments with no excitations in their vicinity have no opportunities to reorganize and are temporarily trapped in their present IS. Single excitations give the system two equally probable options, occupation of *IS*_*A*,1_ or *IS*_*B*,1_. With no other reorganization events in the same vicinity, *IS*_*A*,1_ and *IS*_*B*,1_ both remain open to the system (the rearrangement is reversible), and no real relaxation has occurred. If two excitations coincide in the same vicinity we have four equally probable outcomes: {*IS*_*A*,1_; *IS*_*A*,2_}, {*IS*_*B*,1_; *IS*_*A*,2_}, {*IS*_*A*,1_; *IS*_*B*,2_}, {*IS*_*B*,1_; *IS*_*B*,2_}. Without considering coupling, each possibility is equally weighted, and there is a 75% chance that at least one new IS will be occupied, but the system can still return to its original state. If, in our simple model, we allow a particle rearrangement to induce a strain field^46^ that biases inherent state potentials for nearby particles, then we must consider coupling between excitations. In this case, a *IS*_*A*,1_ → *IS*_*A*,2_ rearrangement in excitation 1 biases the potential of the nearby particles involved in excitation 2, favoring either the *IS*_*A*,1_ or *IS*_*B*,2_ choice of that excitation. Once this biased choice is made for excitation 2, the *IS*_*B*,1_ state is stabilized, and a relaxation has occurred. By this argumentation, *P*_0_ and *P*_1_ are fundamentally distinct from *P*_2_, in that the former two cannot relax, but the latter can. Environments with two nearby excitations behave qualitatively the same as environments with three or more nearby excitations.

To quantify environment populations, we must define a “vicinity” as used in the paragraph above. Assuming that dynamics are influenced primarily by the structure of the first solvation shell,^13,47^ we classify local dynamic environments by the number of molecules within the first shell involved in excitations.

Using measured Φ_0_ values, coordination numbers (*z*), and mild assumptions, we can estimate populations *P*_0_, *P*_1_, and *P*_2_. In Figure 3, we present these population estimates for all systems studied. We arrive at these population estimates by assuming that excitations are randomly distributed in space, that *P*_0_ = 1 at *T* = 0, and that *z* is temperature-independent. Under these assumptions we can write a set of ordinary differential equations that are exact in the limit Φ_0_ → 0: *dP*_0_/*dΦ*_0_ = −(*z* + 1) *P*_0_, where *z* is the coordination number, *dP*_1_/*dΦ*_0_ = (*z* + 1) (*P*_0_ – *P*_1_), and *dP*_2_/*dΦ*_0_ = (*z* + 1) *P*_1_. These equations do not account for correlation effects that come into play at higher Φ_0_ values, but for systems such as those of interest here, with *z* ≈ 12, the deviations are inconsequential. (See Supporting Information for comparison to exact lattice calculations.) Additionally, the bottom panel in Figure 3 compares the estimated environment populations and those directly tabulated from the simulations, showing only minor deviations. Coordination numbers for each system are calculated from simulation as described in Methods and shown in Table 2.

**Figure 3:**
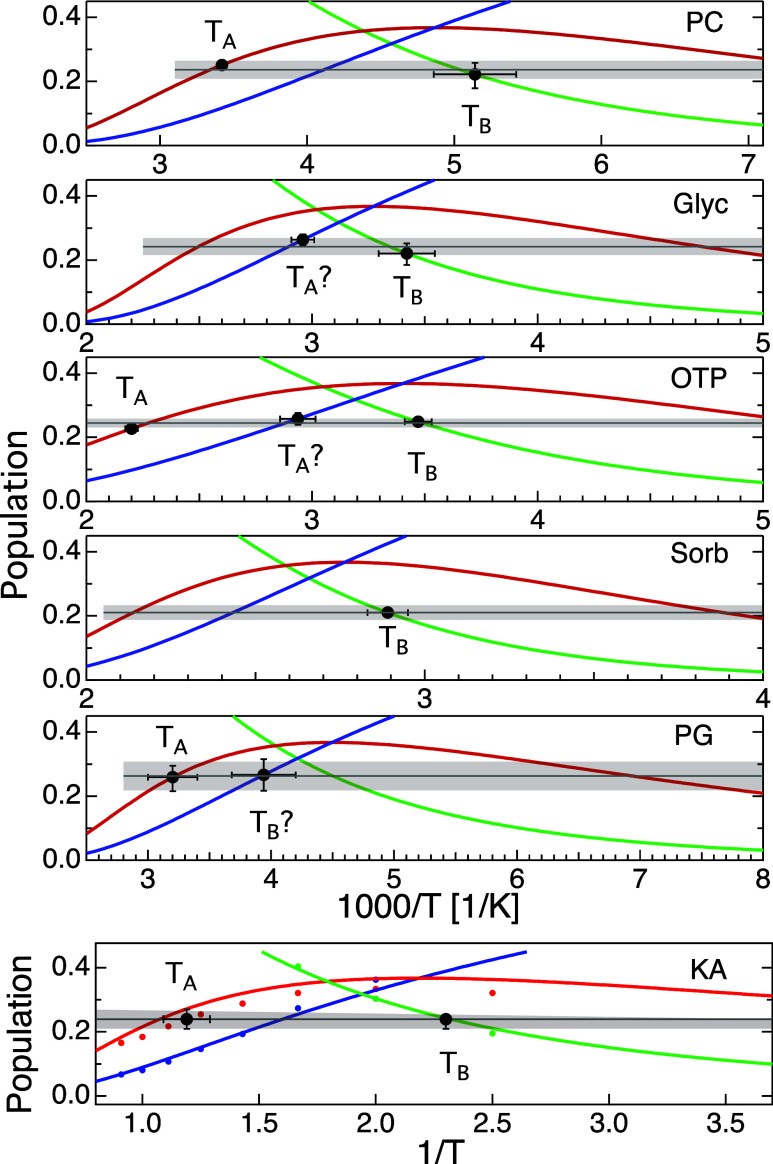
Estimated populations of environments with 0, 1, and ≥2 excitations in the first shell as a function of temperature for liquids indicated and the KA model. Green, red, and blue lines indicate *P*_2_, *P*_1_, and *P*_0_ populations, respectively, identified from excitations defined at 1 ps. Green, red, and blue points in the bottom panel are environmental populations calculated from simulation trajectories. *T*_A_ and *T*_B_ values, where established, are marked by solid black circles and occur where *P*_1_ and *P*_2_, respectively, have a population of 0.24 ± 0.02. Temperatures marked with *T*_A_? or *T*_B_? fall where *P*_0_ = 0.24 ± 0.02, and we suspect that it has been erroneously assigned. The low-temperature limits of all graphs except glycerol correspond roughly to the Vogel temperature, *T*_0_.

The *T*_A_ and *T*_B_ values in Figure 3 and their uncertainties are derived from literature values listed in Table 1. We find it striking that one of the populations *P*_*i*_ has a value 0.24 ± 0.02 at each of these temperatures. In most cases, *P*_1_ and *P*_2_ = 0.24 within uncertainty at *T*_A_ or *T*_B_ respectively. In this figure, vertical error bars at *T*_A_ and *T*_B_ are obtained by projecting temperature uncertainty onto the associated population line.

The colored data points in the bottom panel of Figure 3 show environment populations calculated directly from simulation trajectories. Although excitations are inhomogeneously distributed in the KA model,^44^ our *P*_*i*_ population estimations are quite close to measured values in this system, suggesting that the assumption of randomly distributed excitations is not too severe. Aside from the assumption of random distribution, the populations are estimated with no free parameters.

The probability that a single characteristic temperature for any individual liquid would randomly fall at *P*_*i*_ = 0.24 is approximately equal to the uncertainty in that *P*_*i*_ value (2σ_*i*_) at that temperature, divided by the total range that *P*_*i*_ takes. Accordingly, we estimate the log probability of the null hypothesis for *N* such temperatures as *ln*(*H*_0_) ≈ *N* *ln*[2σ_*i*_/range­(*P*_*i*_)], where we conservatively assign range­(*P*_*i*_) = 0.35 (although *P*_0_ and *P*_2_ range from 0 to 1). *H*_0_ values for individual liquids are modest, but *H*_0_ ≈ 10^–8^ for all systems combined. This strongly supports our initial hypothesis that there is a mechanistic tie between populations of dynamic environments and the mechanistic crossovers at *T*_A_ and *T*_B_. A closer investigation of the data allows us to refine this initial hypothesis.

The data of Figure 3 indicates that mechanistic changes to relaxation occur for each liquid at a specific fraction (0.24) of a given dynamic environment. To us, this suggests that the percolation of dynamically distinct environments underlies these mechanistic changes. Consistent with this idea, we note that for OTP, glycerol, and PG, characteristic temperature values thought to have been *T*_A_ or *T*_B_ have been reported at precisely the temperatures where *P*_0_ = 0.24. If the percolation hypothesis is correct, this would correspond to a solidification temperature where, on a picosecond time scale, a solid-like domain would first percolate. These temperatures are reported in Table 1 under column headings “*T*_A_?” and “*T*_B_?”.

Despite the considerable circumstantial evidence for percolation, 0.24 ± 0.02 is much higher than expected for a site percolation critical fraction (*p*_c_) for amorphous systems with coordination numbers in the range (11–13) for as for systems considered here (see Table 2). For fcc and hcp lattices, which have *z* = 12, *p*_c_ ranges from (0.195 to 0.199). Random-packed systems are expected to have *p*_c_ values equal to or slightly smaller than regular lattices with the same *z*.^48^ We turn to an analysis of the KA model trajectories to test whether the patterns in Figure 3 actually signify percolation events, and if so, what precisely is percolating. Of course, the KA model does not represent the wide range of intermolecular interactions represented by the other liquids, but all these liquids exhibit what appear to be very similar or the same mechanistic crossovers at *T*_A_ and *T*_B_ so it is reasonable to assume that the detailed topology of the PEL landscape may not matter much, and that what matters is there is a landscape with significant barriers. Additionally, we have previously demonstrated that on time scales and length scales associated with interbasin hops, motion in molecular systems can be mapped onto the behavior of simple systems such as the KA model.^16,49^ Below, we test the percolation hypothesis in the KA system by directly calculating percolation thresholds for dynamic environments. As we will see below, analysis of the KA model provides evidence that the high *p*_c_ values inferred from the data in Figure 3 are an artifact of counting both transient and longer-lived dynamic environments when percolation of only the longer-lived environments might be relevant.

### Percolation of Mobile and Immobile Domains

We identify dynamic environments for each A particle in the KA system as follows: First, all A particles are given a label value, *l* = 1 if the particle is involved in an excitation or *l* = 0 if not. First coordination shells are then defined as all A particles within an interaction radius, *R*_AA_ = 1.42 σ, and the environment of the center particle is assigned as *P*_0_, *P*_1_, and *P*_2_ if the sum of *l* values over the central particle and its first shell is 0, 1, or ⩾2, respectively.

Once environments are assigned, we compute the percolation probability for each dynamic environment *P*_0_, *P*_1_, and *P*_2_. Percolation for a given type of environment (e.g., *P*_0_) is defined as when the particles form a system-spanning network composed of continuous connections between particles, fully spanning the periodic simulation box. Connections are contacts between nearest neighbor particles, using the criteria above *R*_AA_ = 1.42 σ to define nearest neighbors. For each simulation snapshot, a value of “1” is assigned if the environment type (e.g., *P*_0_) percolates as defined above, or “0” otherwise; the percolation probability is then the average over the simulation trajectory. Percolation of each frame is determined using a recursive search algorithm of Edvinsson et al.^50^ It is known that percolation behavior follows a “step function”, exhibiting a sharp transition at the percolation threshold “*p*_c_”, with systems percolating/nonpercolating for concentrations above/below *p*_c_. This sharp transition in percolation behavior is only rigorous in the infinite system size limit,^50^ and for finite system sizes (i.e., with periodic boundary conditions) *p*_c_ must be estimated, as the observed percolation transition occurs over a concentration range. For the KA system sizes investigated here, we estimate a few percent uncertainty in determining *p*_c_ due to finite size effects,^51^ which is acceptable for our purposes given the corresponding uncertainties in determining populations *P*_0_, *P*_1_, and *P*_2_.

As a control, we also compute percolation for a random distribution of particles. We randomly assigned excitation labels to varying fractions of A particles and found that each environment percolated at *p*_c_ = 0.2 as expected. When excitations were identified based on particle mobility as described above, we found that domains *P*_0_, *P*_1_, or *P*_2_ percolate with *p*_c_ = 0.14, indicating that excitations are not randomly distributed but form local clusters, consistent with the results of others.^44^ This confirmed that the apparent *p*_c_ = 0.24 value from Figure 3 was aberrant.

Excitations and dynamic environment populations were assigned using a rolling 1.3 ps window, applied at 0.1 ps intervals. We filter the most unstable environment assignments by considering only those dynamic environments that retain the same designation over three consecutive assignment intervals. Applying this criterion, we obtain environment percolation at the expected temperatures. *P*_*i*_ values for persistent environments are shown in Figure 4A. *p*_c_ is exceeded at *T*_A_ for immobile particles, those that persist in *P*_0_ or *P*_1_ domains. Likewise, the population of mobile particles, those that persist in *P*_2_ environments, drops below *p*_c_ at *T*_B_.

**Figure 4:**
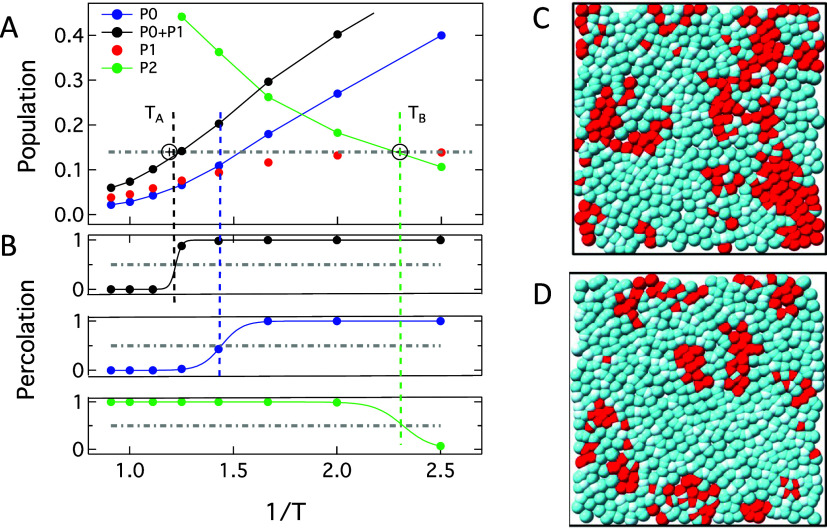
(A) Populations of dynamic environments in the KA system that persist for at least 0.3 ps after being first defined, for a total persistence time of at least 1.6 ps. The open circles with plus signs indicate *T*_A_ and *T*_B_. The observed percolation threshold *p*_c_ = 0.14 is indicated as a gray dashed line. (B) Black, blue, and green circles give the probability that (*P*_0_+*P*_1_), *P*_0_ alone, and *P*_2_ domains that persist for ≈1.6 ps in KA will form percolating clusters. The solid lines are sigmoidal fits and are intended as guides to the eye. (C, D) Percolating and nonpercolating environments. The red particles belong to *P*_2_ environments, while the cyan particles are a collection of those in *P*_0_ and *P*_1_. (C) Example percolating environment for *P*_2_, taken from a *z*-axis slice of a *T* = 0.5 trajectory. (D) Example nonpercolating environment for *P*_2_, taken from a *z*-axis slice of a *T* = 0.4 trajectory.

From top to bottom, the three panels in Figure 4B show percolation probabilities for all slow environments (particles in either *P*_0_ or *P*_1_ environments), solid-like environments *P*_0_, and fluid environments (*P*_2_). We see that (*P*_0_ + *P*_1_) percolates at *T*_A_, rather than simply *P*_1_ as suggested in Figure 3. The percolation of solid-like environments occurs at a temperature intermediate between *T*_A_ and *T*_B_, and the bottom panel shows that mobile *P*_2_ domains cease percolating at *T*_B_.

Figure 4C and D provide examples of percolating and nonpercolating environments for *P*_2_, above and below *T*_B_ ≈ 4.3 at *T* = 0.5 and *T* = 0.4, respectively. The images are 2D slices along the *z*-axis of ∼1.3σ in depth from our 3D trajectories. All particles from the slice were projected onto a single plane for visualization.

Considering only longer-lived dynamic environments leads to reasonable values for *p*_c_ and percolation at the expected temperatures, but there is a notable difference in the ratio of *P*_0_ to *P*_1_ populations defined over shorter times and slightly longer periods, with *P*_0_/*P*_1_ increasing significantly for longer-lived environments. To gain insight into this, we consider the persistence characteristics of each environment. We define a time-dependent environment population *P*_*i*_(Δ*t*) as the fraction of particles that continuously meet the criteria for inclusion in envronment *P*_*i*_ for a time interval Δ*t*, and η_*i*_(Δ*t*) = *P*_*i*_(Δ*t*)/*P*_*i*_(0) where *P*_*i*_(0) = *P*_*i*_ as defined above. Figure 5A–C shows these lifetime plots for A particles of the KA model, and panel D shows average environment lifetimes, calculated as first moments of η_*i*_(Δ*t*). *P*_1_ environment assignments are short-lived, with characteristic lifetimes (τ_*P*1_) that are only weakly temperature dependent and less than 300 fs at all temperatures. This profile suggests that *P*_1_ environments are found primarily at interfaces between *P*_0_ and *P*_2_ domains. The temperature dependences of τ_*P*0_ and τ_*P*2_ suggest that these environments form bulk domains. These would be solid-like and fluid-like, respectively. With lifetimes no larger than the inverse relaxation attempt frequency, it is understandable that *P*_1_ environments on their own do not seem to have an impact on dynamic mechanism. By contrast, *P*_0_ and *P*_2_ lifetimes are such that significant fractions of these environments persist long enough to influence local relaxation attempts.

**Figure 5:**
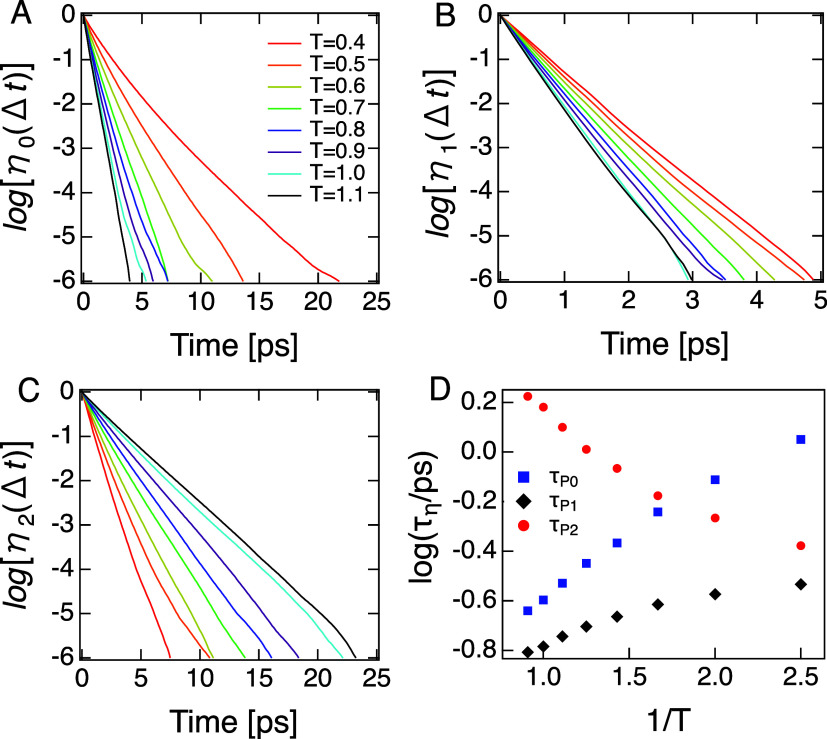
(A–C) Temporal persistence profiles of dynamic environments and temperatures indicated for the KA model. (D) Environment lifetimes, defined as the first moment of the distributions in panels A–C.

## Discussion

Above, we have presented an analysis of experimental and simulation data suggesting that the crossovers in relaxation mechanism at *T*_A_ and *T*_B_ arise from the percolation of distinct dynamic environments in the liquid on a picosecond time scale. Others have also found that percolation appears to play a role in thermodynamic^52^ and dynamic^53–58^ properties of liquids and glasses. Of these previous reports, the works of Novikov et al.^53^ and Gao et al.^58^ are most directly relevant to the present study.

Gao et al.^58^ recently showed that β_JG_ and α relaxation, measured through the shear modulus, occur respectively on the shortest time scale at which mobile particles begin to percolate and immobile particles cease to percolate for a given temperature. In their simulations, they defined all particles as either mobile or immobile depending on their displacements over the time window in which the material relaxation was probed.

Novikov et al.^53^ showed that experimental values for *T*_B_ (the mode-coupling *T*_c_) and the Vogel temperature, *T*_0_ (far below *T*_g_) are consistent with percolation of a mobile domain at *T*_0_ and percolation of a solid-like domain at *T*_B_, assuming percolation thresholds of 15%, and classifying all particles as in solid-like or mobile environments depending on whether they exhibit picosecond-time scale mean-squared displacements in the upper half or lower half of an assumed log-normal distribution.

On their face, the Gao^58^ and Novikov^59^ interpretations of mobile particle percolation appear inconsistent. Together, they seem to suggest that β_JG_ relaxation should occur on a picosecond time scale at *T*_0_, whereas τ_β,JG_ is much slower, usually on the order of μs to ms at the much higher temperature, *T*_g_. Our analysis resolves this issue and is consistent with results from both papers.

Given that Novikov et al.^59^ considered large excursions on a ps time scale, they would have considered particles in *P*_1_ and *P*_2_ environments as mobile. Since their analysis dealt with low temperatures, *P*_1_ environments would be three to four times more prevalent than *P*_2_. On the other hand, Gao et al.^58^ would have considered only particles in persistent *P*_2_ environments as mobile since they performed their analysis on time scales roughly (3 to 5) orders of magnitude larger, and the lifetime of *P*_1_ environments is so short. Our analysis shows that Gao’s mobile particles (those in *P*_2_), percolate on a ps time scale at *T*_B_. Below this temperature, *P*_2_ percolation would occur on increasingly long time scales, consistent with Gao’s results. Likewise, Novikov’s mobile particles in *P*_1_ environments appear to cease percolating near *T*_0_ for most of the systems we have analyzed in Figure 3. *P*_1_ populations cross the apparent percolation line near the lowest temperatures plotted in these systems, which, in all cases except glycerol, correspond to *T*_0_.

We likewise attribute the incompatibility of the Gao and Novakov analysis of immobile particle percolation to the assignment of immobile particles. Novikov et al. inferred the presence of immobile particles and estimated that they should percolate near *T*_B_. As we show in Figures 3 and 4, solid-like domains do percolate near, but at a slightly higher temperature than *T*_B_. In other published work, this higher temperature percolation event seems to have been mistaken for *T*_B_ or *T*_A_ in OTP, glycerol, and propylene glycol (see Table 1). Gao et al. assign mobile and immobile particles to be consistent with a two-Gaussian van Hove function, similar to what we have done, but on a longer time scale, so their assignments should correlate closely to the immobile (*P*_0_+*P*_1_) assignments in our work, which, on a picosecond time scale percolate at *T*_A_. As Dyre points out,^60^ this percolation, which Gao showed occurs at longer times for lower temperatures, is essentially a facilitation, allowing α relaxation to occur while only sampling the smallest barriers to reorganization.

In addition to the evidence presented above for the proposed percolation scenario, we discuss below how this proposed scenario, along with the assumption that excitations are required for relaxation, leads us to plausible pictures for mechanistic crossovers in liquid dynamics. To reiterate from previous sections, *P*_0_ environments are solid-like and unable to relax, and *P*_1_ environments support only single IB, reversible transitions. In *P*_2_ environments, reversible IB transitions can be entropically trapped by a quasi-synchronous and nearby IB transition, leading to nonreversing relaxation events.

At *T* > *T*_A_ in the proposed scenario, both *P*_0_ and *P*_1_ exist but neither forms extended domains. Figure 5D shows that both these environments are transient on the time scale of α relaxation at *T* > *T*_A_, which is also evinced by the Arrhenius temperature dependence and lack of obvious dynamic heterogeneity at the τ_α_ time scale. The existence of slow environments that persist on a ps time scale at *T* > *T*_A_ is demonstrated by the bimodal structure of the van Hove function at 1 ps.^18^ We understand these results from *P*_0_ and *P*_1_ existing primarily as individual loci or small droplets surrounded by *P*_2_ at *T* > *T*_A_. Intimate proximity with *P*_2_ environments means that *P*_1_ or *P*_0_ particles will quickly become involved in an excitation, leading to the interconversions *P*_0_ ↔ *P*_1_ ↔ *P*_2_ on the time scale τ_Φ_, where τ_Φ_ < 0.25 ⟨τ_α_⟩ for *T* ≥ *T*_A_.^16^ Interconversion of these environments on such a short time scale would obfuscate dynamic heterogeneity effects, and α relaxation would *appear* to be homogeneous and collisional.

At *T* < *T*_A_, signatures of dynamic heterogeneity and excitation-facilitated hops become detectable,^5,6,61^ which we understand as a consequence of increased persistence of slow domains (*P*_0_ + *P*_1_) concomitant with their percolation. The appearance of extended *P*_0_ + *P*_1_ environments is consistent with expectations of a spinodal at this temperature.^62^

At all temperatures *T* > *T*_B_, where extended *P*_2_ environments exist, multiple pathways will be available to each particle for interbasin transits in those environments. Thus, particles in extended *P*_2_ domains undergoing an interbasin transit will likely have a particle in their first shell involved in a nearly independent but simultaneous interbasin transit. In these cases, the local structure would rearrange somewhat within the period of the barrier transit. If close enough, those nearby rearrangements would tend to alleviate local stress and reduce the extent of displacement required to establish a new inherent state. This accounts for the reduced hop length (σ_IB_) observed at elevated temperatures in actual liquids.^18^ This scenario allows us to define small-step diffusion as instantaneously facilitated relaxation dynamics and understand why small-step diffusion is observed only at *T* > *T*_B_,^6,63,6,63^ where extended *P*_2_ domains exist. The existence of extended *P*_2_ domains only at *T* > *T*_B_ further explains the observation that saddle points are encountered when quenching simulations to identify inherent states from this temperature regime,^64^ and with a localization transition (loss of access to saddle points) at *T* < *T*_B_.

Below the temperature where *P*_0_ percolates, solid-like domains should begin to persist long enough to impact dynamics. Accordingly, there is a shift in the temperature dependence of the Brillouin line at precisely this temperature in PC.^65^ Additionally, this temperature seems to have been mistakenly identified as *T*_A_ or *T*_B_ in OTP, glycerol, and PG. Finally, two-level systems are known to disappear in glasses prepared with a sufficiently low equilibrium structure.^66^ This is predicted in the scenario proposed here when *P*_1_ drops below the detection limit in the equilibrated glass at some temperature well below *T*_B_. Finally, we note in passing that Figure 3 suggests *P*_1_ environments may drop below a percolation threshold at the Kauzman temperature. Investigating the implications (if any) of this on dynamics will be addressed at a later time.

## Summary

We provide evidence that the percolation of mobile and immobile domains on a picosecond time scale drives mechanistic crossovers in relaxation at *T*_A_ and *T*_B_. We also show that such percolation events provide mechanistic explanations for a wide range of the dynamic phenomena documented at these temperatures. Discriminating between three distinct picosecond-time scale dynamic environments also allows us to resolve apparent contradictions between results of previous works that also propose dynamic percolation.^58,59^ Assuming that this percolation hypothesis is substantially correct, we are left with two compelling questions: (1) Why do we find percolation for mobile and immobile regions only when we define dynamic environments as those that persist for at least 300 fs after they have been identified, and (2) Why do all the experimental liquids seem to behave just as the KA system in Figure 3, with apparent *p*_c_ values of 0.24 when dynamic environment populations are estimated from Φ_0_?

The KA model cannot reproduce all the dynamic features that arise from the detailed chemical structures of molecular liquids. On the other hand, we have previously given examples where KA maps onto molecular systems when time scales and length scales are properly averaged,^16,18,49^ the dynamic features we are concerned with here—excitations and interbasin hops—appear to provide that averaging to a large extent. Regarding the environment persistence question, ≈1 ps, where Φ­(*t*) transitions to a slower decay (see Figure 2) is the shortest time at which it is physically meaningful to define an excitation or a dynamic environment. Likely, some of the excitations identified at this time are merely rare deformations of unusually elastic inherent states. This would lead to a transient misidentification of some dynamic environments, which would be rectified with a slightly extended persistence criterion.

Fully addressing these questions will require analyzing atomistic simulation trajectories for at least each of the liquids in Figure 3, as we have done for the KA system. This is presently out of reach for two reasons. The first is that *T*_A_ and *T*_B_ are not known for the force field Hamiltonians of the liquids other than the Kob-Andersen model. There is no question that *T*_A_ and *T*_B_ from simulations of propylene carbonate, glycerol, sorbitol, and OTP will vary from the values obtained from the liquids themselves. For example, in PC we have found that even a very sophisticated, polarizable ab initio force field cannot reproduce the experimental scattering function *F*(*q*,*t*) with enough fidelity for correspondence of excitation populations between experiment and simulation.^51^ This precludes any correspondence between simulation thresholds (e.g., percolation) and experimental thresholds of *P*_0_, *P*_1_, and *P*_2_ as determined in Figure 3. Determination of *T*_A_ and *T*_B_ for each liquid force field/Hamiltonian would require extensive simulation beyond that presented in this work. The second, more compelling reason is that precisely how one defines excitations in a molecular liquid simulation has yet to be worked out.

Finally, the transition between the picture suggested by Figures 3 and 4 requires specific lifetimes for the three dynamic environments. If excitations are prone to cluster, then *P*_0_ and *P*_2_ will form bulk domains and *P*_1_ would be found mostly at interfaces as seems to be the case here. If this is the case, then the persistence time scales come down to the size and fractal dimension of the domains, and the fundamental time scale for excitation mobility. The latter should be several multiples of the zero-crossing time of the particle velocity autocorrelation time, and should not depend strongly on temperature or molecular system. If the characteristic temperatures *T*_A_ and *T*_B_ are defined by some function of the size and fractal dimension of the *P*_0_ and *P*_2_, then these are constrained to maintain similarity in some gauge for all systems. We look forward to exploring these questions.

## Supplementary Material



## Data Availability

The neutron scattering data is available by request to the NIST Center for Neutron Science. Simulation trajectories and the code to analyze the neutron and simulation data are available at the request of the corresponding author.

## Notes

Details on fitting the QENS data, finding *T*_A_ for the Kob-Andersen (KA) Lennard-Jones model system, selecting coordination numbers for OTP, and using the intermediate scattering function in simulation to identify excitations (PDF)
