# Supplementary material for: A Case for Dynamic Percolation Underlying Mechanistic Crossovers in the Relaxation of Liquids
Source: J Phys Chem B. 2025 Jun 17;129(26):6620–31. doi: 10.1021/acs.jpcb.5c01033 (PMC12235649; doi:10.1021/acs.jpcb.5c01033)
Supplement: Supplementary file 1 [file jp5c01033_si_001.pdf]

Supporting Information for "A Case for Dynamic Percolation Underlying Mechanistic Crossovers in the Relaxation of Liquids"

Marcus T. Cicerone,*,† Jessica Z. Dixon,† John P. Stoppelman,‡ Kelly Badilla-Nunez,¶ and Jesse G. McDaniel*,†

†*Department of Chemistry and Biochemistry, Georgia Institute of Technology 950 Atlantic Drive, Atlanta, GA 30332, USA*

‡*Institute of Chemistry, Academia Sinica, Taipei 115, Taiwan*

¶*School of Chemical and Biochemical Engineering, Georgia Institute of Technology 900 Atlantic Drive, Atlanta, GA 30332, USA*

E-mail: cicerone@gatech.edu; jesse.mcdaniel@chemistry.gatech.edu

Modeling QENS Data

In this work, we use an explicitly heterogeneous dynamics model to describe the QENS data. We account for distinct types of motion expected in amorphous systems: over-damped vibration (IS motion), hops (IB transitions), and homogeneous diffusion. Based on ultrafast optical experiments performed by us,[1] and on work by Vispa et al.,[2] we use Lorenzians to represent each of the three types of motion considered here. Vispa et al.,[2] found that a triple Lorentzian function provided significantly better fits for $S(q, E)$ of a molecular liquid than common models of similar complexity containing functional forms such as KWW and Gaussian, and much better fits than 2-component models. Likewise, we find that a three-

Lorenzian model fits scattering data well for each of the liquids analyzed here. Consistent with this, we observe three exponential relaxation processes for PC in time-domain optical Kerr effect data covering similar time and lengthscales to those considered here. Accordingly, we use the following model for $S(q,E)$:

$$\begin{aligned} S(q,E) =& (1-\tilde{\Phi}(E))L_D \otimes [(1-a_v)\,\delta(E) + a_v L_v] \\ &+ \tilde{\Phi}(E) L_D \otimes [(1-a_v)\,\delta(E) + a_v L_v] \otimes [(1-a_h)\,\delta(E) + a_h L_h] \end{aligned} \qquad (1)$$

where $L_i = \Gamma_i \pi^{-1}(E^2+\Gamma_i^2)^{-1}$, $\otimes$ is the convolution operator, and the convolutions are over frequency (energy). $\Gamma_D = D_T q^2$ where D_T is the diffusion coefficient, and a_v and a_h are respectively, q-dependent scattering amplitudes from vibration (IS motion) and hopping (IB crossing). Anticipating that these two will be localized modes with Gaussian distributions of displacements,[3] we assume the functional form:

$$a_i(q) = c_i\{1 - exp[-(\pi\sigma_i q)^2]\} \qquad (2)$$

where the σ_i represents the characteristic lengthscales of motion for mode i.

The two terms in Eq (2) account for two dynamically different classes of molecules. We assume that all molecules undergo both diffusion and over-damped vibrations at all times. Accordingly, both terms in Eq. (2) include diffusive and vibrational motion. We also assume that, up to a given time, only some fraction (Φ) of molecules have participated in collective hopping motion (h) associated with IB transitions. These are accounted for in the second term of Eq. (2).

Data are first fitted in the frequency domain to obtain q-dependent characteristic frequencies Γ for each mode of motion. Data are then transformed to the time domain $F(q,t)$ and fitted to the time-domain representation of Eq. (2) to obtain $\Phi(t)$:

$$F(q,t) = e^{-t/\tau_D}[1 + a_v(e^{-t/\tau_v} - 1)] \times [(1-\Phi(t)) + \Phi(t)(1 + a_h(e^{-t/\tau_h} - 1))] \qquad (3)$$

where $\tau_i = \hbar/\Gamma_i$. In the regime $t\Gamma_D < \hbar < t\Gamma_h \leq t\Gamma_v$, we can ignore terms involving τ_v and τ_h, and Eq. (3) reduces essentially to

$$F(q) = (1 - \Phi(t))e^{-(q\,\pi\,\sigma_v)^2} + \Phi(t)e^{-(q\,\pi\,\sigma_h)^2} \tag{4}$$

We have used to Eq. (3) when only short time ($\approx 1\,ps$) is of interest.[4] In either case, $\Phi(t)$ represents the fraction of molecules that have hopped (undergone an IB crossing) up to time t. Bearing in mind that hops constitute significantly larger excursions than vibrations, it is clear that until a molecule executes a hop, the q-dependence of its scattering signature will be characteristic of only small lengthscale motion. Once a hop occurs, that signature will change to a larger lengthscale for all subsequent times.

The QENS data, as obtained in the frequency domain, is a convolution of the molecular scattering function and instrument resolution

$$S_{expt}(q, E) = S_{molecular}(q, E) \otimes R(q, E) \tag{5}$$

where $R(q, E)$ is instrumental resolution. The model was convolved with the instrument response function when fitting in the frequency domain. The frequency-domain convolution operation is simple multiplication in the time domain:

$$F_{expt}(q, t) = F_{molecular}(q, t) \times R(q, t) \tag{6}$$

where $R(q, t)$ is the Fourier transform of $R(Q, E)$. Thus, the molecular response was directly deconvolved from the overall signal as $F_{molecular} = F_{expt}/R$.

$S(q, E)$ obtained at 4 K (or its Fourier transform) was used as the resolution function $R(q, E)$. Scattering from samples at 4 K was compared with standard vanadium and found to contain no broadening from, e.g. methyl group rotation, etc., so these data sets were deemed acceptable for normalizing $F(q, t)$. The fact that coherent scattering is only about 5% of

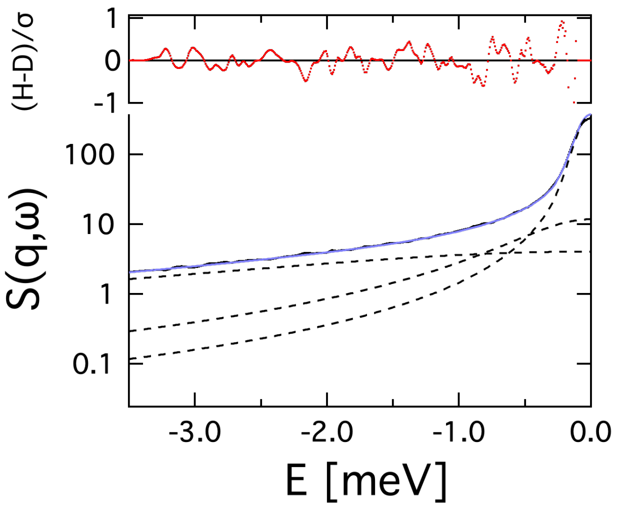

Figure S1: Fits to $S(q = 0.8\text{Å}^{-1}, E)$ of PC, measured at T=300 K, using Eq. (2). The solid black line is the QENS data, $S(q, E)$. The solid blue line is the fit. The dashed lines are the fit components from three Lorenzians with $\Gamma_D = 0.05$ meV, $\Gamma_{IB} = 0.55$ meV, and $\Gamma_{IS} = 2.9$ meV. Fit residuals, normalized to uncertainty in the data points, are shown in the upper part of the figure.

the total scattering in these samples ameliorates the complications arising from coherent scattering when base temperature data is used as a resolution for higher temperatures.

In both frequency-domain and time-domain fitting, we used an iterative simulated annealing algorithm implemented in PyMC (pymc.io), a Bayesian analysis framework, to find optimized fit parameters. In the frequency domain, we found values for Γ_i at each average q value. Figure S1 shows the fit of Eq. (2) to quasielastic neutron scattering (QENS) from PC at 300 K and q= 0.8 Å^{-1}. The model is convolved with the instrument resolution. The upper trace shows fit residuals (H-D), normalized by the standard deviation (sd) at each data point. These normalized residuals are randomly distributed between -1 and 1 for all data reported, as in Fig. S1.

We used Eq. (3) to fit the time domain data shown in Fig.S2, finding optimized parameters for $\Phi(t)$ and from Eq. (1), c_i and σ_i. Figure S2A shows fits of Eq. (3) to $F(q, t)$ from PC at 300 K at a series of times from 600 fs to 6 ps. Figure S2(B-D) shows the time dependence of Φ, σ_{IS}, and σ_{IB} respectively at the temperatures indicated.

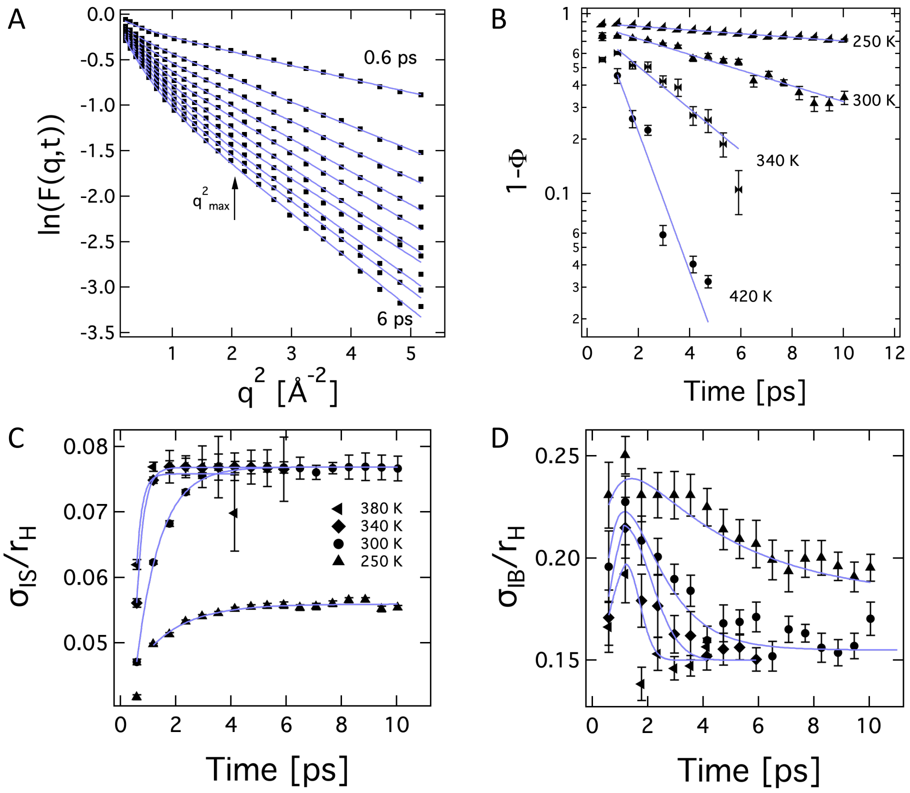

Figure S2: $F(q,t)$ from PC at 300 K and fits to Eq. (4). (A)Fits to $F(q,t)$ calculated at times ranging from 0.6 ps to 6 ps in increments of 0.6 ps. Solid lines are fits to Eq. (4). The uncertainties in the data are approximately the size of the symbols. (B) Time dependence of Φ, derived from fits of Eq. (4) to F(q,t) for temperatures indicated. Dashed lines are exponential decays. (C-D) Time dependence of $\tilde{\sigma}_{IS}$ and $\tilde{\sigma}_{IB}$ derived from fits of Eqs. (3) and (4) to F(q,t) data in the range (250–380) K for PC. The solid lines are guides to the eye. Error bars indicate uncertainties in parameters at one standard deviation.

Φ, σ_{IS}, and σ_{IB} each show time dependence consistent with their assignments. σ_{IS} reaches an asymptote consistent with a localized process. σ_{IB} behaves non-monotonically as expected if hop events have a non-zero probability of reversal. Φ increases monotonically from ≈ 0 to 1.

Molecular Dynamics Simulations used to Determine Coordination Numbers

PC and sorbitol simulation consisted of 400 and 200 molecules, respectively, initially constructed using Packmol.[5] NPT simulations at 1 bar were performed at each temperature using a Monte Carlo Barostat and were run for 10 ns, and subsequent NVT simulations were run for 100 ns. All simulations were run using a 1 fs timestep using the GPU-accelerated OpenMM software.[6] The *ab initio*, polarizable SAPT-FF force field was used for propylene carbonate, while the CHARMM36 force field was used for sorbitol.[7,8] The procedure for constructing the force field for propylene carbonate was described elsewhere.[9] A dual-Langevin thermostat was used for treating the Drude oscillator degrees of freedom for the propylene carbonate simulations, with a 1 ps^{-1} friction coefficient.[10] A standard Langevin thermostat was used for sorbitol, also with a 1 ps^{-1} friction coefficient. The particle-mesh Ewald method (PME) was used for modeling electrostatics in all simulations, and a 1.4 nm cutoff was used for handling van der Waals (VDWs) interactions.[11]

For glycerol, molecular dynamics simulations were performed as described in ref.[12] using the GROMACS simulation package[13] and a modified OPLS forcefield.[14] Nine independent systems of 425 molecules each were simulated with 2 fs integration time steps and particle-mesh Ewald summations.[11] Simulations were equilibrated at 500 K, cooled at a rate of 0.1 K/ps to the appropriate temperature for data collection, and allowed to equilibrate for an additional 2 ns. During cooling, a Parrinello–Rahman barostat[15] was used, and the temperature was maintained using velocity rescaling.

OTP was simulated using the Lewis-Wahnström (LW) 3-site model.,[16] and MD simulations of 200 OTP molecules were run using the GROMACS software package.[17] The LINCS algorithm was used to keep bonds and angles constrained.[18] NPT simulations were run at 1 bar for 5 ns using the Parrinello-Rahman barostat,[19] and subsequent NVT simulations were then performed for 5 ns using a Nosé-Hoover thermostat with a time constant of 1 ps.[20] These LW OTP simulations yielded center-of-mass z values of 16.3, 15.6, and 15.2 at 300 K,

400 K, and 500 K, respectively, in agreement with LW model calculations from Boue et al.[21]

KA Simulation Details

Table 1 below gives details on the molecular dynamics trajectories generated for the KA system.

Table 1: Details of molecular dynamics trajectory lengths and trajectory output resolution for KA simulations at varying temperature in reduced temperature (T) and time (τ) units.

T	trajectory length	trajectory frame output
0.4	5000τ	0.05τ
0.5	12000τ	0.05τ
0.6	6000τ	0.05τ
0.7	3000τ	0.05τ
0.8	300τ	0.05τ
0.9	300τ	0.05τ
1.0	300τ	0.05τ
1.1	300τ	0.05τ

T_A for KA

For the KA model, T_A was determined from the temperature dependence of α relaxation times. τ_α were assigned as the time of the 1/e point of $F_s(q,t)$ at q_{max} for A particles. The τ_α vs 1/T data from which T_A is derived is shown Fig. S3A. From τ_α we identify $T_A = 0.84 \pm .0.9$. Above T_A we find Arrhenius behavior for τ_α, but a quadratic temperature dependence in E_A for $T < T_A$.

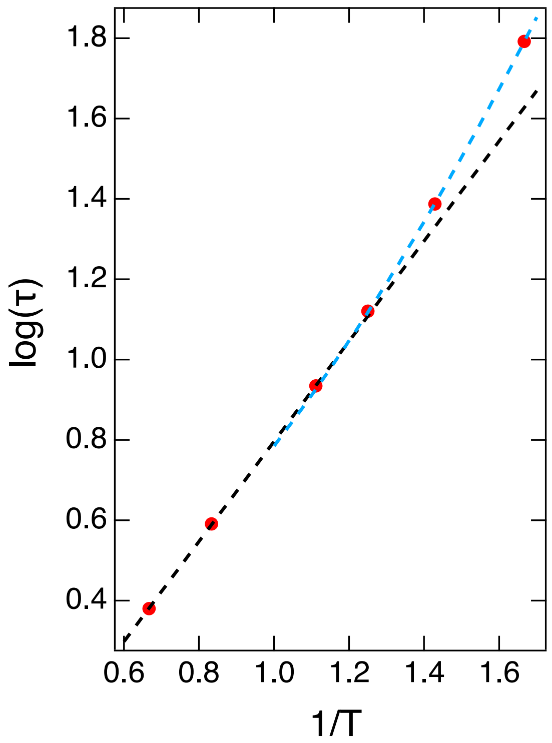

Figure S3: Relaxation times for A particles in the KA system, determined through $F_s(q,t)$ The black dashed line is a linear fit, and the blue dashed line is quadratic in $(1/T - 1/T_A)$ with $T_A = 0.84 \pm 0.09$.

Coordination Number for OTP

Using z in the range (15 to 16) results in P_0, P_1, and P_2, values far from 0.24. Rather, we find agreement between the behavior of OTP and the other molecules if we use a z value for OTP phenyl rings rather than molecular centers of mass, and this is what we have reported in the main manuscript. We use a phenyl ring z value of 11.5, which is intermediate between values obtained in LW OTP and atomistic OTP simulations performed by Hung et al.[22]

Figure S4 shows that characteristic temperatures do not fall at iso-population points when using the OTP molecular center of mass coordination number, z=15.5 to calculate P_0, P_1, and P_2 as they do when we use the phenyl ring coordination number, as in Fig. 3B of the main text. This suggests that it matters only whether a phenyl group in the first shell of another phenyl group is involved in an excitation. We can understand this if we assume that excitations involve only a small number of atoms. For example, if two OTP molecules are positioned so that their central rings are adjacent, an excitation involving a few atoms in the

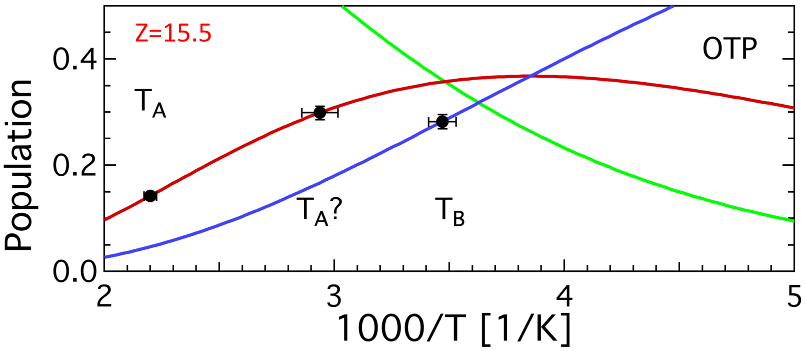

Figure S4: Estimated populations of environments with 0, 1, and ≥ 2 excitations in the first shell as a function of temperature for OTP, using the molecular center of mass coordination number, z = 15.5.

central ring of molecule 1 is likely to impact the dynamic environment of the central ring of molecule 2, whereas an excitation on a distal ring of molecule 1 would be unlikely to do so. This sub-molecular hypothesis is consistent with what is known of these phenomena from LJ simulations and with comparisons of H-atom and COM intermediate scattering functions.[9] In LJ simulations, only a few particles are simultaneously involved in an excitation, and LJ particles are more representative of atoms than of entire molecules. Also, analysis of H-atom and center-of-mass intermediate scattering functions in glycerol[12] show that COM motion qualifying as hops is relatively rare compared to hop prevalence from incoherent H-atom motion.

Difficulties in Using Simulation to Quantify Excitations in Molecular Systems

There are presently two major difficulties in using simulations to quantify excitations in molecular systems. On issue, as we have recently discussed,[9] is that motion involved in excitations includes low-frequency intra-molecular vibration and intermolecular motion. The accuracy of standard force fields may not be sufficient to describe these motions in quantitative agreement with experiment. The other issue is related to complexities in defining excitations that arise in the presence of intra-molecular bonds.

The first issue is exemplified for propylene carbonate in Fig. S5, with data taken from ref.[9] Similar results can be found in ref.[12] In both cases, the simulation predicts decay of $F_s(q,t)$ much faster than that measured experimentally at $q \geq 1\,\text{Å}^{-1}$, and additional nuances of the experimental shape are not recovered. In comparing $F(q,t)$ in Fig. S5, note that the ordinate axis extends to -4 for simulation and only to -3 for the experimental result. Note also that the propylene carbonate force field was developed based on *ab initio* calculations of intermolecular interactions, and yet still exhibited discrepancy compared to experiment. It is thus an important research goal to improve the simulation/force field description of motions involved in excitations. We speculate that a higher accuracy treatment of intramolecular vibrations (i.e. bonded force field terms), and/or inclusion of nuclear quantum effects could improve the description of excitations. With regard to the latter, high-frequency intramolecular vibrations must certainly be treated quantum mechanically, yet excitations involve the lower frequency modes.[9] The importance of nuclear quantum effects in describing excitations thus remains an open question.

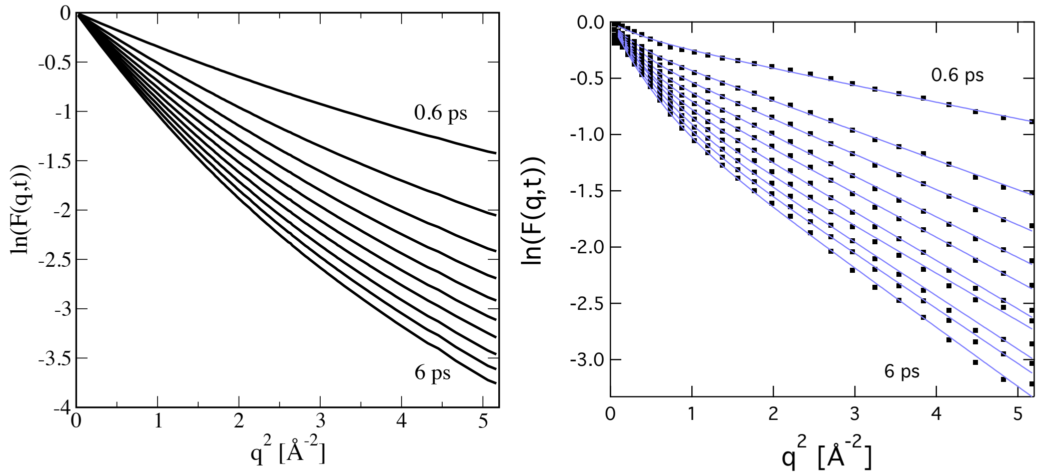

Figure S5: Intermediate scattering functions for propylene carbonate at 300K. Left: $F(q,t)$ calculated from simulation trajectories as described in ref.[9] Right: Points are $F(q,t)$ measured using QENS, and lines are fits to the data using equation (1) in the main manuscript.

The second issue, *a priori* identification of excitations in molecular systems is touched on above in the description of finding an appropriate z value for OTP. Repeating that text:

Figure S4 shows that characteristic temperatures do not fall at iso-population points when using the OTP molecular center of mass coordination number, z=15.5 to calculate P_0, P_1, and P_2 as they do when we use the phenyl ring coordination number, as in Fig. 3B of the main text. This suggests that it matters only whether a phenyl group in the first shell of another phenyl group is involved in an excitation. We can understand this if we assume that excitations involve only a small number of atoms. For example, if two OTP molecules are positioned so that their central rings are adjacent, an excitation involving a few atoms in the central ring of molecule 1 is likely to impact the dynamic environment of the central ring of molecule 2, whereas an excitation on a distal ring of molecule 1 would be unlikely to do so. This sub-molecular hypothesis is consistent with what is known of these phenomena from LJ simulations and with comparisons of H-atom and COM intermediate scattering functions.[9] In LJ simulations, only a few particles are simultaneously involved in an excitation, and LJ particles are more representative of atoms than of entire molecules. Also, analysis of H-atom and center-of-mass intermediate scattering functions in glycerol[12] show that COM motion qualifying as hops is relatively rare compared to hop prevalence from incoherent H-atom motion.

Environment Estimates

The ODEs used to estimate populations P_0, P_1, and P_2 ignore lattice crowding effects, but the associated errors are small. Below we plot the integrated ODEs and an exact solution for an FCC lattice. We follow that with the Python code used to generate these values.

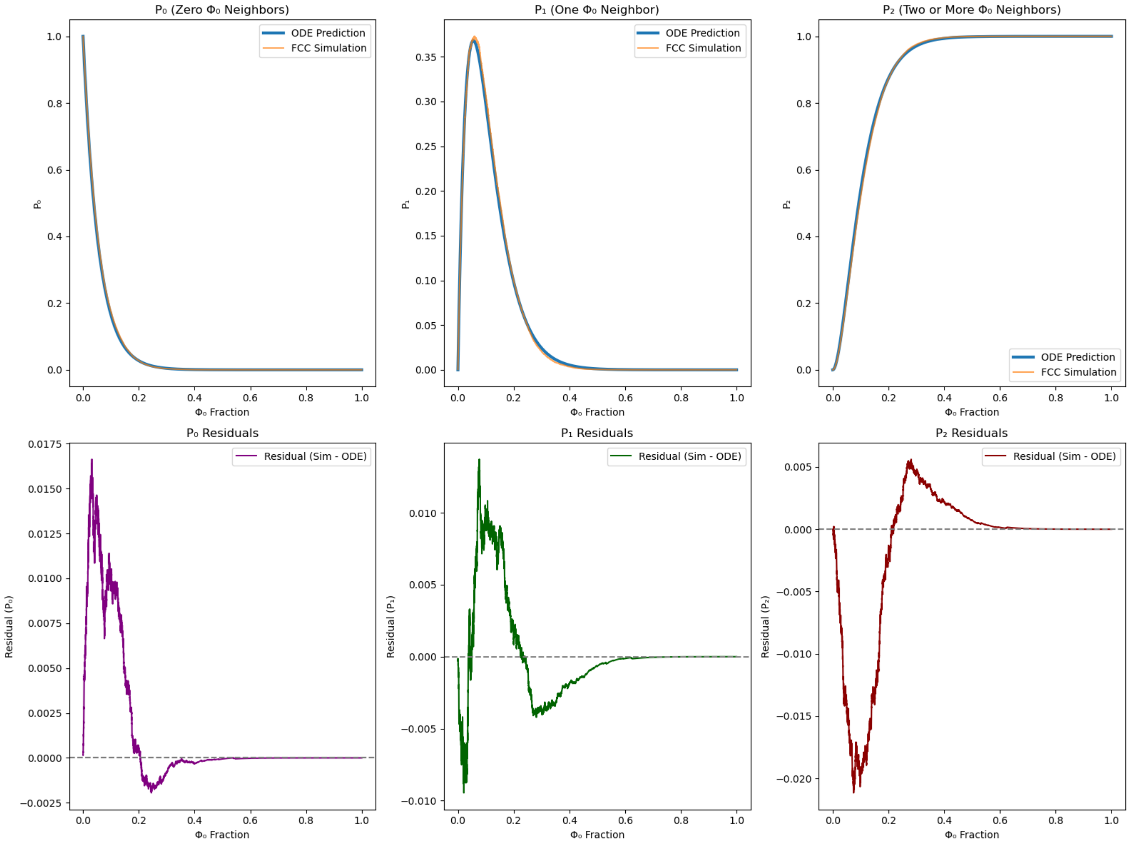

Figure S6: Top row: Envrionment population calculations using the approximate ODEs, and exact calculations on a FCC lattice. Bottom row: Residuals between the ODE and FCC calculations.

```
import numpy as np

import matplotlib.pyplot as plt

from scipy.spatial import cKDTree



# ---------------------------

# FCC LATTICE GENERATION FUNCTION

# ---------------------------
```

```python
def generate_fcc_lattice(n_cells):
    """Generate FCC lattice points within n_cells x n_cells x n_cells unit cells.
    """
    a = 1.0 # Lattice constant (arbitrary units)
    points = []

    # FCC basis vectors
    basis = np.array([[0, 0, 0],
                      [0.5, 0.5, 0],
                      [0.5, 0, 0.5],
                      [0, 0.5, 0.5]])

    for x in range(n_cells):
        for y in range(n_cells):
            for z in range(n_cells):
                origin = np.array([x, y, z]) * a
                for b in basis:
                    points.append(origin + b * a)

    return np.array(points)

# ----------------------------
# GENERATE FCC LATTICE
# ----------------------------

n_cells = 20 # Size of the lattice (20x20x20 FCC unit cells)
lattice_points = generate_fcc_lattice(n_cells)
N_sites = lattice_points.shape[0]
```

```
# Build KD-tree to find nearest neighbors efficiently
tree = cKDTree(lattice_points)
neighbors_list = tree.query_ball_tree(tree, r=1.01) # Slightly above NN distance

# Remove self from neighbor lists and store
neighbors_array = [np.array([n for n in neighbors if n != i]) for i, neighbors in
    enumerate(neighbors_list)]
z = np.mean([len(n) for n in neighbors_array]).astype(int) # Should be ~12 in FCC

# ----------------------------
# INITIALIZATION
# ----------------------------

n_steps = 50000 # Number of 0 markers to add

site_marker = np.zeros(N_sites, dtype=int) # 0 if no marker, 1 if marker
neighbor_count = np.zeros(N_sites, dtype=int) # Number of marked neighbors

P0_sim = []
P1_sim = []
P2_sim = []
Phi0_fraction = []

# ----------------------------
# LATTICE SIMULATION (True FCC neighbors)
# ----------------------------

for step in range(n_steps):
    # Randomly select a site to add 0
```

```
    candidates = np.where(site_marker == 0)[0]
    if len(candidates) == 0:
        break
    selected = np.random.choice(candidates)
    site_marker[selected] = 1

    # Update neighbors
    neighbors = neighbors_array[selected]
    neighbor_count[neighbors] += 1

    # Calculate populations
    P0 = np.sum(neighbor_count == 0) / N_sites
    P1 = np.sum(neighbor_count == 1) / N_sites
    P2 = np.sum(neighbor_count >= 2) / N_sites

    P0_sim.append(P0)
    P1_sim.append(P1)
    P2_sim.append(P2)
    Phi0_fraction.append(np.sum(site_marker) / N_sites)

# ----------------------------
# ODE MODEL SOLUTION
# ----------------------------

Phi0_ode = np.linspace(0, np.max(Phi0_fraction), 1000)

P0_ode = np.exp(-(z + 1) * Phi0_ode)
P1_ode = (z + 1) * Phi0_ode * np.exp(-(z + 1) * Phi0_ode)
P2_ode = 1 - P0_ode - P1_ode
```

References

(1) Bender, J. S.; Zhi, M.; Cicerone, M. T. The polarizability response of a glass-forming liquid reveals intrabasin motion and interbasin transitions on a potential energy landscape. *Soft Matter* **2020**, *16*, 5588 – 5598.

(2) Vispa, A.; Busch, S.; Tamarit, J. L.; Unruh, T.; Fernandez-Alonso, F.; Pardo, L. C. A robust comparison of dynamical scenarios in a glass-forming liquid. *Physical Chemistry Chemical Physics* **2015**, *18*, 3975–3981.

(3) Rahman, A.; Singwi, K. S.; Sjölander, A. Theory of Slow Neutron Scattering by Liquids. I. *Physical Review* **1962**, *126*, 986 – 996.

(4) Cicerone, M. T.; Zhong, Q.; Tyagi, M. Picosecond Dynamic Heterogeneity, Hopping, and Johari-Goldstein Relaxation in Glass-Forming Liquids. *Physical Review Letters* **2014**, *113*, 117801.

(5) Martínez, J. M.; Martínez, L. Packing optimization for automated generation of complex system's initial configurations for molecular dynamics and docking. *Journal of Computational Chemistry* **2003**, *24*, 819–825.

(6) Eastman, P.; Swails, J.; Chodera, J. D.; McGibbon, R. T.; Zhao, Y.; Beauchamp, K. A.; Wang, L. P.; Simmonett, A. C.; Harrigan, M. P.; Stern, C. D.; Wiewiora, R. P.; Brooks, B. R.; Pande, V. S. OpenMM 7: Rapid development of high performance algorithms for molecular dynamics. *PLoS Computational Biology* **2017**, *13*, e1005659.

(7) McDaniel, J. G.; Schmidt, J. R. Physically-motivated force fields from symmetry-adapted perturbation theory. *J. Phys. Chem. A* **2013**, *117*, 2053–2066.

(8) Huang, J.; Mackerell, A. D. CHARMM36 all-atom additive protein force field: Validation based on comparison to NMR data. *Journal of Computational Chemistry* **2013**, *34*, 2135–2145.

(9) Stoppelman, J. P.; McDaniel, J. G.; Cicerone, M. T. Excitations follow (or lead?) density scaling in propylene carbonate. *The Journal of Chemical Physics* **2022**, *157*, 204506.

(10) Lamoureux, G.; Roux, B. Modeling induced polarization with classical Drude oscillators: Theory and molecular dynamics simulation algorithm. *Journal of Chemical Physics* **2003**, *119*, 3025–3039.

(11) Essmann, U.; Perera, L.; Berkowitz, M. L.; Darden, T.; Lee, H.; Pedersen, L. G. A smooth particle mesh Ewald method. *The Journal of Chemical Physics* **1995**, *103*, 8577–8593.

(12) Cicerone, M. T.; Averett, D.; Pablo, J. J. d. The role of hopping on transport above Tc in glycerol. *Journal of Non-Crystalline Solids* **2015**, *407*, 118–125.

(13) Hess, B.; Kutzner, C.; Spoel, D. v. d.; Lindahl, E. GROMACS 4: Algorithms for Highly Efficient, Load-Balanced, and Scalable Molecular Simulation. *Journal of Chemical Theory and Computation* **2008**, *4*, 435–447.

(14) Averett, D.; Cicerone, M. T.; Douglas, J. F.; Pablo, J. J. d. Fast relaxation and elasticity-related properties of trehalose- glycerol mixtures. *Soft Matter* **2012**, *8*, 4936–4945.

(15) Parrinello, M.; Rahman, A. Polymorphic transitions in single crystals: A new molecular dynamics method. *Journal of Applied Physics* **1981**, *52*, 7182–7190.

(16) Lewis, L. J.; Wahnström, G. Molecular-dynamics study of supercooled *ortho*-terphenyl. *Physical Review E* **1994**, *50*, 3865.

(17) Abraham, M. J.; Murtola, T.; Schulz, R.; Páll, S.; Smith, J. C.; Hess, B.; Lindah, E. Gromacs: High performance molecular simulations through multi-level parallelism from laptops to supercomputers. *SoftwareX* **2015**, *1-2*, 19–25.

(18) Hess, B. P-LINCS: A Parallel Linear Constraint Solver for Molecular Simulation. *Journal of Chemical Theory and Computation* **2007**, *4*, 116–122.

(19) Parrinello, M.; Rahman, A. Polymorphic transitions in single crystals: A new molecular dynamics method. *Journal of Applied Physics* **1998**, *52*, 7182.

(20) Hoover, W. G. Canonical dynamics: Equilibrium phase-space distributions. *Physical Review A* **1985**, *31*, 1695.

(21) Boué, L.; Hentschel, H. G. E.; Ilyin, V.; Procaccia, I. Statistical Mechanics of Glass Formation in Molecular Liquids with OTP as an Example. *The Journal of Physical Chemistry B* **2011**, *115*, 14301–14310.

(22) Hung, J.-H.; Simmons, D. S. Do String-like Cooperative Motions Predict Relaxation Times in Glass-Forming Liquids? *The Journal of Physical Chemistry B* **2019**, *124*, 266 – 276, doi: 10.1021/acs.jpcb.9b09468.
